# Supplementary material for: Next-generation proteomics for quantitative Jumbophage-bacteria interaction mapping
Source: Nat Commun. 2023 Aug 24;14:5156. doi: 10.1038/s41467-023-40724-w (PMC10449902; doi:10.1038/s41467-023-40724-w)
Supplement: Supplementary file 1 — Supplementary Information [file 41467_2023_40724_MOESM1_ESM.pdf]

Next-generation proteomics for quantitative Jumbophage-bacteria interaction mapping

Andrea Fossati[1,2,3†], Deepto Mozumdar[4†], Claire Kokontis[4], Melissa Mèndez-Moran[5], Eliza Nieweglowska[5], Adrian Pelin[1,2,3], Yuping Li[4], Baron Guo[4], Nevan J. Krogan[1,2,3], David A. Agard[5], Joseph Bondy-Denomy[4*] and Danielle L. Swaney[1,2,3*]

[1]J. David Gladstone Institutes, San Francisco, 94158, California, USA.
[2]Quantitative Biosciences Institute (QBI), University of California San Francisco, San Francisco, 94158, California, USA.
[3]Department of Cellular and Molecular Pharmacology, University of California San Francisco, San Francisco, 94158, California, USA.
[4]Department of Immunology and Microbiology, University of California San Francisco, San Francisco, 94158, California, USA.
[5]Department of Biochemistry, University of California San Francisco, San Francisco, 94143, California, USA.

*Corresponding author(s). E-mail(s): Joseph.Bondy-Denomy@ucsf.edu; danielle.swaney@ucsf.edu;
Contributing authors: andrea.fossati@gladstone.ucsf.edu; deepto.mozumdar@ucsf.edu; claire.kokontis@ucsf.edu; melissa.mendez@ucsf.edu; eliza@msg.ucsf.edu; adrian.pelin@ucsf.edu; yuping.li@ucsf.edu; baron.guo@ucsf.edu; nevan.krogan@ucsf.edu; agard@msg.ucsf.edu;
†These authors contributed equally to this work.

Supplementary Information

Supplementary Figures

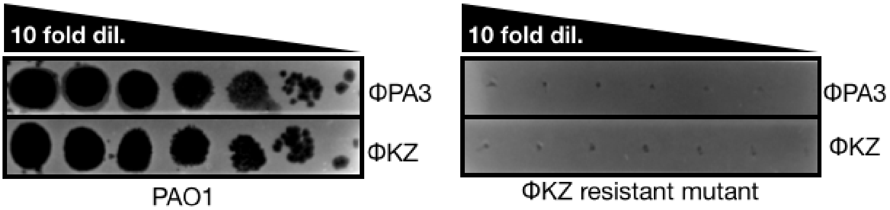

Supplementary Fig. S 1. Plaque assays with Jumbophages ϕKZ and ϕPA3 spotted in 10-fold serial dilutions on a lawn of PAO1 (WT) or PAO1 (ϕKZ resistant mutant) strains. Clearings represent phage replication.

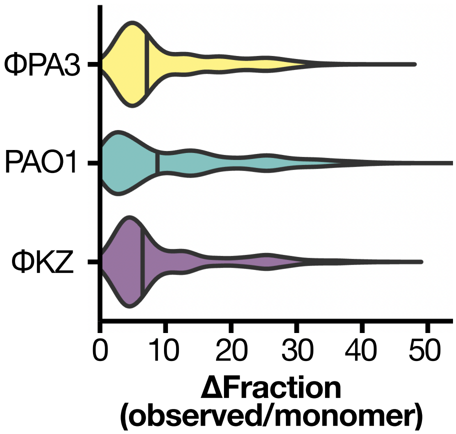

Supplementary Fig. S 2. Violin plot showing the distance between the observed protein SEC peak and their predicted molecular weight expressed as fraction number for the single-peak proteins. Black line represents the mean.

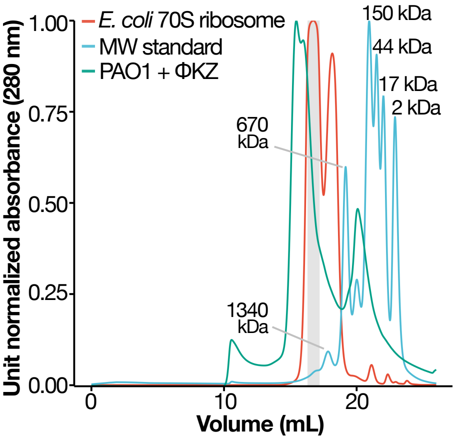

Supplementary Fig. S 3. Ribosome absorption profile and MW from SEC Chromatographic traces collected at 280 nm for the purified 70S ribosome (red), the protein mixture used as molecular weight standards (cyan) and the ϕKZ infected sample (green). The grey area shows the fractions used for the ribosomal crosslinking experiment.

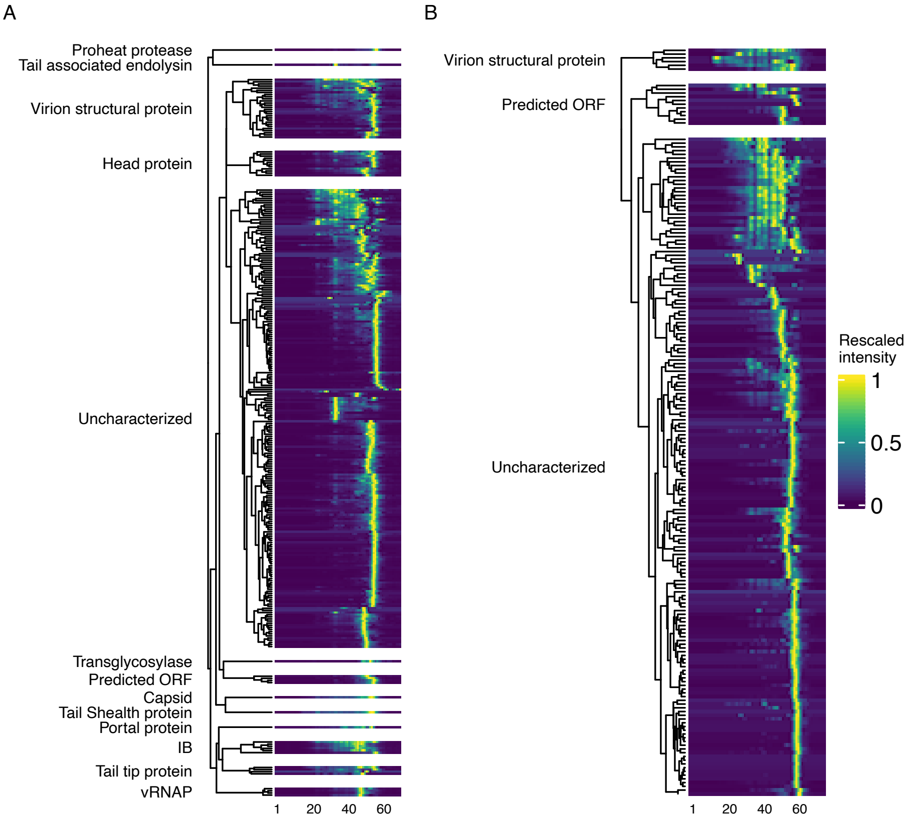

Supplementary Fig. S 4. Distribution of the KZ-like phages proteomes into discrete assemblies A-B. Coelution heatmap for all the phage proteins identified in ϕKZ (**A**) and ϕPA3 (**B**). The dendrogram branches are labelled based on manual literature curation for the corresponding proteins in the peak group. Color represents the unit-rescaled intensity. X axis represent the fraction number.

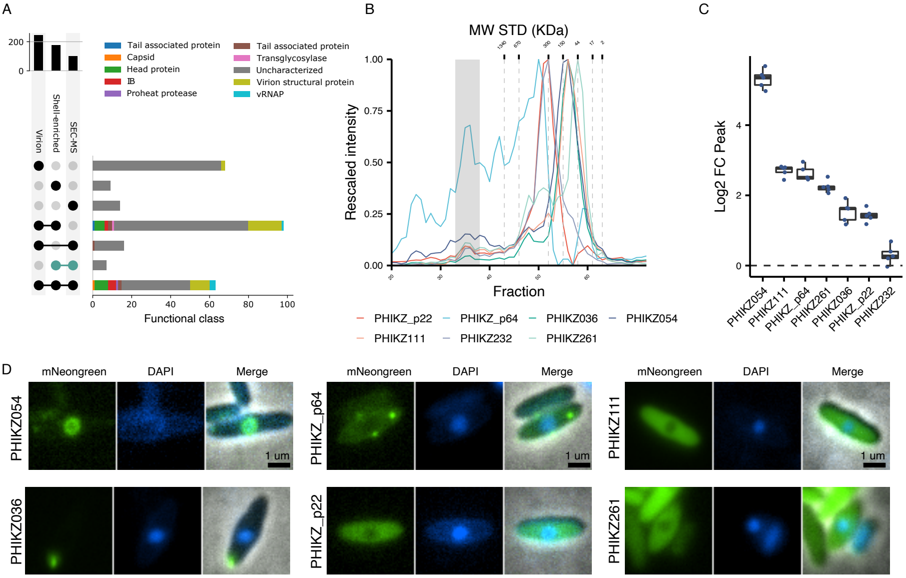

Supplementary Fig. S 5. Predicted ϕKZ shell proteome A. Upset plot showing the overlap between the ϕKZ virion enrichment, shell enrichment and SEC-MS experiment. Green barbell shows the retained putative shell proteome **B**. Coelution profile for main shell protein (gp54) and its predicted interaction partners. **C**. Enrichment (Log2FC) over average protein intensity for the proposed shell proteins (n=10 independent fractions for the shell associated peak). The box boundaries show the interquantile range (IQR) and its whiskers 1.5×IQR. Black lines represents the mean. **D**. Fluorescent microscopy of proposed shell associated proteins.

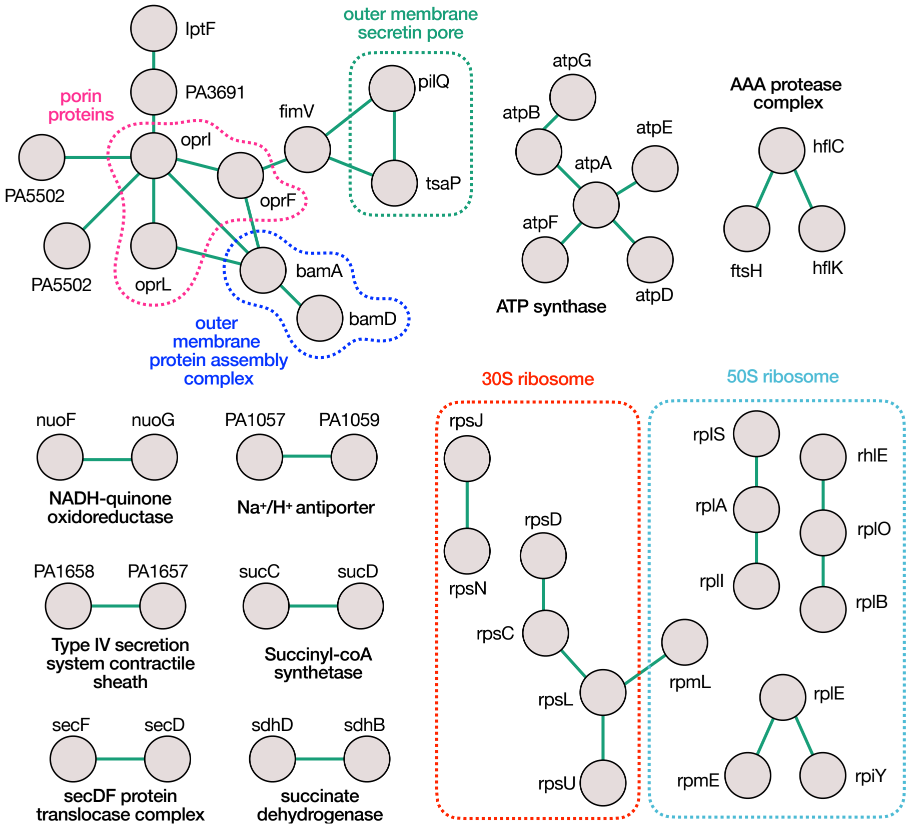

Supplementary Fig. S 6. Know protein complexes recovered by SEC-XL MS Interaction network derived from the heterolinks detected at 5% CSM-FDR for reported complexes in *Pseudomonas*. Text label shows the complex for the interacting proteins.

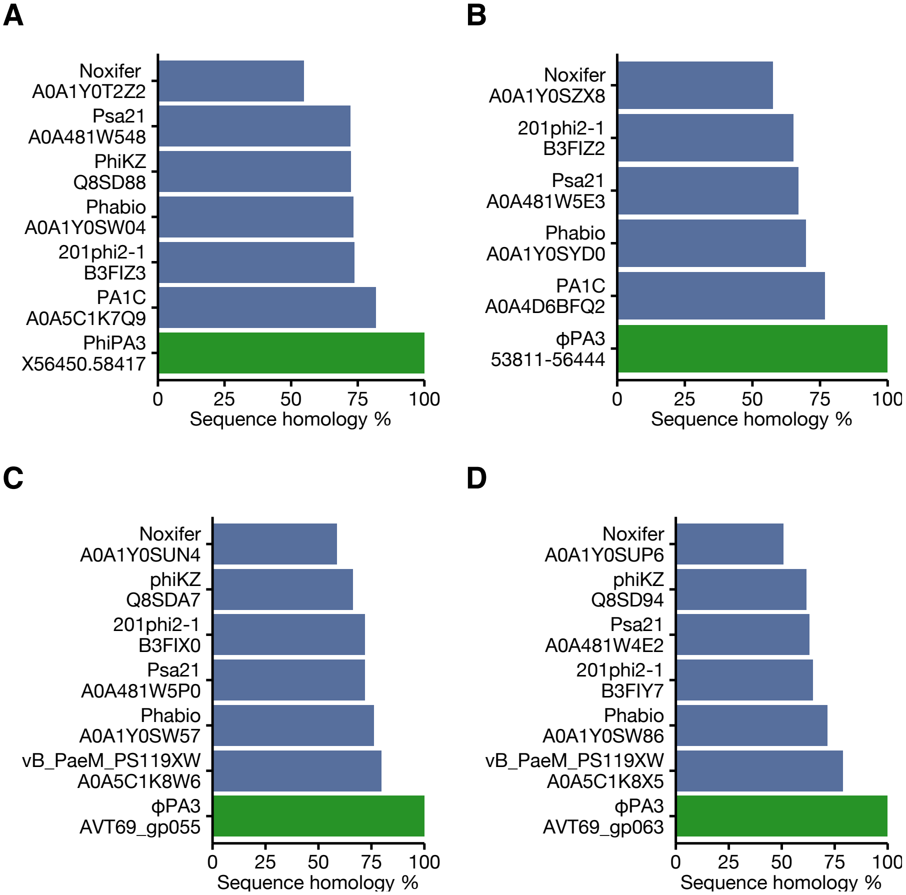

Supplementary Fig. S 7. Alignment of ϕPA3 56450-58417 interactors with other Jumbophage proteins A-D. Barplot showcasing the sequence homology between ORF 56450-58417 (**A**), 53811-56444 (**B**), gp55 (**C**) and gp63 (**D**) to other *Pseudomonas* phages protein. ϕPA3 proteins are highlighted in green. X axis shows the percentage of sequence homology.

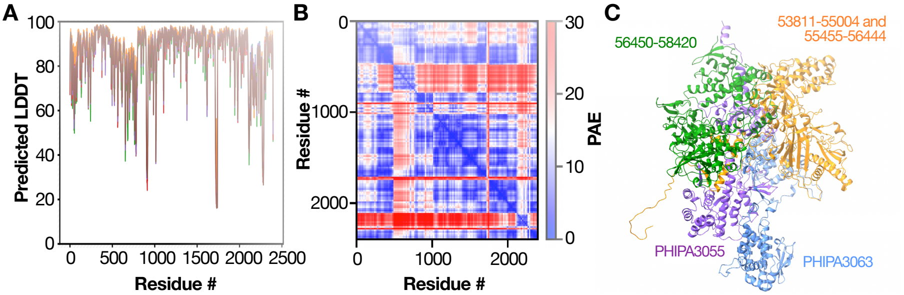

Supplementary Fig. S 8. Prediction of ϕPA3 non virion associated RNA structure A. Per-residue local confidence (pLDDT) versus sequence (X axis). Different line colors represents the different AF2 model **B**. Predicted alignment error (PAE) heatmap **C**. Structure of best scoring model (iPTM + TM = 0.861)

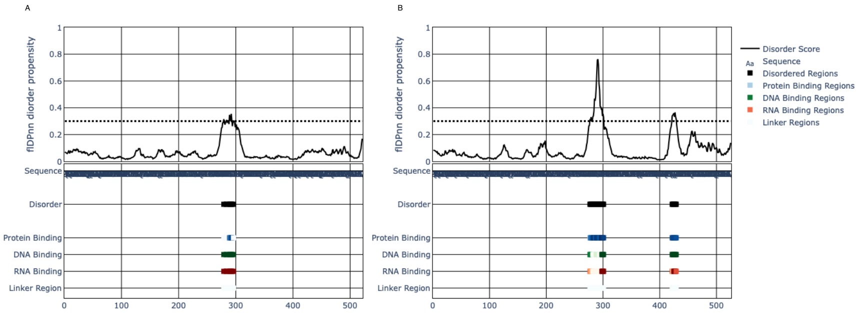

Supplementary Fig. S 9. Intrinsically disordered region in ϕKZ gp68 and ϕPA3 gp63 Prediction of disordered regions using flDPnn[1]. X axis represent sequence, while different rows shows different local predicted properties between ϕKZ gp68 (**A**) and ϕPA3 gp63 (**B**)

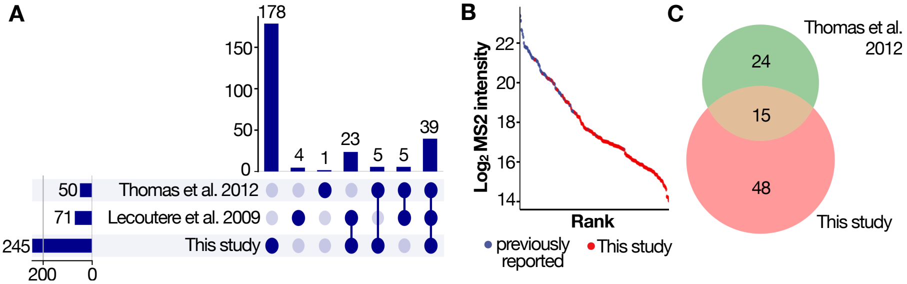

Supplementary Fig. S 10. Benchmark of purified virion MS versus other reported studies of ϕKZ head proteins. **A**. Upset plot showing the overlap in protein IDs between this study, [2] and [3] **B**. Distribution of intensities for virion proteins identified in this study. Blue proteins were previously identified while red proteins are novel virion proteins from this study. **C**. Venn diagramm of semi-tryptic peptides detected in this study versus [2]

Supplementary tables

Table 1. Plasmid sequences utilized in this study

Plasmid Name	Source	Benchling File Link
pHERD30T–PHIKZ030–3xFLAG	This study	https://benchling.com/s/seq-qNAdL4Mf5ZiP66IucsEf
pHERD30T–PHIKZ086 (p29)–3xFLAG	This study	https://benchling.com/s/seq-H7Row3A9kbabSgW4d6Qr
pHERD30T–PHIKZ089–mNeonGreen	[4]	https://benchling.com/s/seq-kqx3tErqsu8D5CvZd4nt
pHERD30T–PHIKZ089–3xFLAG	This study	https://benchling.com/s/seq-ODqqsHuzriBb0fhio4TU
pHERD30T–PHIKZ090–mNeonGreen	[4]	https://benchling.com/s/seq-cvRIWvzJwH1Us1lvRSZJ
pHERD30T–PHIKZ090–3xFLAG	This study	https://benchling.com/s/seq-Iid5LZaAbF4IkCLuvkNO
pHERD30T–PHIKZ092–3xFLAG	This study	https://benchling.com/s/seq-489AypdKLmUvtAvtzuwj
pHERD30T–PHIKZ093–mNeonGreen	[5]	https://benchling.com/s/seq-1HniLlMgETrwE2ynoIO8
pHERD30T–PHIKZ093–3xFLAG	This study	https://benchling.com/s/seq-XQcKRXCfJmmcRAyGv9n2
pHERD30T–PHIKZ094–3xFLAG	This study	https://benchling.com/s/seq-BHUnSQdmkfAQApcv9xb9
pHERD30T–PHIKZ095–mNeonGreen	[4]	https://benchling.com/s/seq-K4ALrCk2b4fKyJiT7Wbl
pHERD30T–PHIKZ095–3xFLAG	This study	https://benchling.com/s/seq-Djykfs89Un8hXVabKvyB
pHERD30T–PHIKZ097–mNeonGreen	[4]	https://benchling.com/s/seq-A3EOVIWVMpLyO8y4UCuz
pHERD30T–PHIKZ097–3xFLAG	This study	https://benchling.com/s/seq-0Ztpv7X9iCiTSHjBpZGy
pHERD30T–PHIKZ129–3xFLAG	This study	https://benchling.com/s/seq-L74jt2SEBzUDK3SdTnCw
pHERD30T–PHIKZ153–3xFLAG	This study	https://benchling.com/s/seq-Ldk4eIxLeczNyhz1IWjH
pHERD30T–PHIKZ157–3xFLAG	This study	https://benchling.com/s/seq-IOoQjItGC2eKY7AHR4z8
pHERD30T–PHIKZ162–mNeonGreen	[4]	https://benchling.com/s/seq-l5LYnMqE2wn8E6q50Jpv
pHERD30T–PHIKZ162–3xFLAG	This study	https://benchling.com/s/seq-PZ9WBxqMbpkx60G8EyBt
pHERD30T–PHIKZ163–3xFLAG	This study	https://benchling.com/s/seq-H7rQcemwWwP77Q4QGz0R
pHERD30T–PHIKZ177–3xFLAG	This study	https://benchling.com/s/seq-jEg2bRmaqUoMjCcNHJ3u
pHERD30T–PHIKZ184–3xFLAG	This study	https://benchling.com/s/seq-jhtlyHMlh5hjSbfa2jki
pHERD30T–PHIKZ203–3xFLAG	This study	https://benchling.com/s/seq-8wqZr6Nml3k6h9oQuhhh
pHERD30T–PHIKZ244–3xFLAG	This study	https://benchling.com/s/seq-AyIPGvLQaL5XVyIg6ZqN
pHERD30T–PHIKZ303–3xFLAG	This study	https://benchling.com/s/seq-9xuMji34NZeSXxATzYD5
pHERD30T–mNeonGreen-3xFLAG	This study	https://benchling.com/s/seq-S1xoRXIFUot06gVkXhuh

Table 2. Primers utilized in this study

Primer Name	Sequence
PHIKZ030_F	GAGATATACATACCCATGGGATCTGATAAGATGGCTCGCTATAATGATCCGATGGCAC
phiKZ030_R	TCTTTGTAGTCCATCGAGCCACCGCCACCGCTTAATCCTACACCGTCGTAAGTTACCCG
PHIKZ086_F	GAGATATACATACCCATGGGATCTGATAAGATGTCTCATGTTAAAGAAGTAGATGTCGAA
PHIKZ086_R	GTCTTTGTAGTCCATCGAGCCACCGCCACCCATTGCATAACCACGTTTAAAGTCATC
PHIKZ092_F	ATACCCATGGGATCTGATAAGATGACTAATAATTCATTTAGTCATTCGGTCTTACTTGAA
PHIKZ092_R	GTCTTTGTAGTCCATCGAGCCACCGCCACCCCTTACCTGGGAGGGATTATTTACTTGTTC
PHIKZ094_3xF_Fwd	ACCCATGGGATCTGATAAGAATTCGAGCTCATGAACTTAAATAAGTTACTTGATAGTTTA
PHIKZ094_3xF_Rvs	GTAGTCCATCGAGCCACCGCCACCCTCGAGTTGGCCATATCTTTCTAATTCC
PHIKZ129_F	GAGATATACATACCCATGGGATCTGATAAGATGGCTATACCAGGGGTTAATGAGGG
PHIKZ129_R	GTCTTTGTAGTCCATCGAGCCACCGCCACCTCCAATCTCATCTGATTCAGTTGACTCGTC
PHIKZ153_F	TACATACCCATGGGATCTGATAAGATGTTTAACATCCATAAATACGCTAAAAGTATTTCA
PHIKZ153_R	GTCTTTGTAGTCCATCGAGCCACCGCCACCTCCAATGATTTCACGAAGAATATCTTCAGT
PHIKZ157_F	GATATACATACCCATGGGATCTGATAAGATGTACAACTTGAAAGGTTTTTACGAGTATCC
PHIKZ157_R	GTCTTTGTAGTCCATCGAGCCACCGCCACCACCAAATTGTTCTACACTCTTACGAGTTAC
PHIKZ163_3xF_Fwd	ACCCATGGGATCTGATAAGAATTCGAGCTCATGTCCACATATGCAGAACTATTAATTACC
PHIKZ163_3xF_Rvs	GTAGTCCATCGAGCCACCGCCACCCTCGAGAATACCTTCATTCTCATAATGAGTAATGCA
PHIKZ177_3xF_Fwd	ACCCATGGGATCTGATAAGAATTCGAGCTCATGTTCTGTAAAGAAACCCAAGCT
PHIKZ177_3xF_Rvs	GTAGTCCATCGAGCCACCGCCACCCTCGAGATTAGAAACTTCTTCTTCGATTTCAACATC
PHIKZ184_F	GAGATATACATACCCATGGGATCTGATAAGATGTCTAAACCTAATCTTGAGTGGTTAGC
PHIKZ184_R	GTCTTTGTAGTCCATCGAGCCACCGCCACCATTACCACTATAGGTACCTTCAGTACCTTG
PHIKZ203_F	TATACATACCCATGGGATCTGATAAGATGATAGACTCATTTAGGAAGCTAATGGGCTCGC
PHIKZ203_R	GTCTTTGTAGTCCATCGAGCCACCGCCACCAAACCGCATATATGCGGGCTGATATGGTTT
PHIKZ244_F	ATACCCATGGGATCTGATAAGATGCCAACTTATATTGAATTAATCAATATTGTCAATGAT
PHIKZ303_3xF_Fwd	ACCCATGGGATCTGATAAGAATTCGAGCTCATGAATAAAATGCTAAACTTCCTAAACCGT
PHIKZ303_3xF_Rvs	GTAGTCCATCGAGCCACCGCCACCCTCGAGCTGGTAGTTTTCATTACCCATTGCTAA
pHERD30T-mNG-3xF_F	GGTGGCGGTGGCTCGATGG
pHERD30T-mNg-3xF_R	CTTATCAGATCCCATGGGTATGTATATCTCCTTCTTAAAGTTAAACAAAATTATTTCTAG

Supplementary References

[1] Hu, G. *et al.* flDPnn: Accurate intrinsic disorder prediction with putative propensities of disorder functions. *Nature Communications* **12** (1), 4438 (2021). URL https://doi.org/10.1038/s41467-021-24773-7. https://doi.org/10.1038/s41467-021-24773-7 .

[2] Thomas, J. A. *et al.* Extensive proteolysis of head and inner body proteins by a morphogenetic protease in the giant Pseudomonas aeruginosa phage ϕKZ. *Molecular Microbiology* **84** (2), 324–339 (2012). https://doi.org/10.1111/j.1365-2958.2012.08025.x .

[3] Lecoutere, E. *et al.* Identification and comparative analysis of the structural proteomes of phiKZ and EL, two giant Pseudomonas aeruginosa bacteriophages. *Proteomics* **9** (11), 3215–3219 (2009). https://doi.org/10.1002/pmic.200800727 .

[4] Li, Y. *et al.* A family of novel immune systems targets early infection of nucleus-forming jumbo phages. *bioRxiv* 2022.09.17.508391 (2022). URL http://biorxiv.org/content/early/2022/09/18/2022.09.17.508391.abstract. https://doi.org/10.1101/2022.09.17.508391 .

[5] Guan, J. *et al.* Bacteriophage genome engineering with CRISPR-Cas13a. *Nature microbiology* **7** (12), 1956–1966 (2022). https://doi.org/10.1038/s41564-022-01243-4 .
