# Supplementary material for: Next-generation proteomics for quantitative Jumbophage-bacteria interaction mapping
Source: Nat Commun. 2023 Aug 24;14:5156. doi: 10.1038/s41467-023-40724-w (PMC10449902; doi:10.1038/s41467-023-40724-w)
Supplement: Supplementary file 3 — Description of Additional Supplementary Files [file 41467_2023_40724_MOESM3_ESM.pdf]

Description of Additional Supplementary Files

File Name: Supplementary Movie 1
Description: Timelapse analysis of gp36.

File Name: Supplementary Movie 2
Description: Timelapse analysis of gp64.
