# Supplementary material for: Next-generation proteomics for quantitative Jumbophage-bacteria interaction mapping
Source: Nat Commun. 2023 Aug 24;14:5156. doi: 10.1038/s41467-023-40724-w (PMC10449902; doi:10.1038/s41467-023-40724-w)
Supplement: Supplementary file 6 — Reporting Summary [file 41467_2023_40724_MOESM6_ESM.pdf]

nature portfolio

Corresponding author(s): Danielle Swaney, Joe Bondy-Denomy

Last updated by author(s): Jul 20, 2023

Reporting Summary

Nature Portfolio wishes to improve the reproducibility of the work that we publish. This form provides structure for consistency and transparency in reporting. For further information on Nature Portfolio policies, see our Editorial Policies and the Editorial Policy Checklist.

Statistics

For all statistical analyses, confirm that the following items are present in the figure legend, table legend, main text, or Methods section.

n/a	Confirmed	
☐	☒	The exact sample size (*n*) for each experimental group/condition, given as a discrete number and unit of measurement
☐	☒	A statement on whether measurements were taken from distinct samples or whether the same sample was measured repeatedly
☐	☒	The statistical test(s) used AND whether they are one- or two-sided<br>*Only common tests should be described solely by name; describe more complex techniques in the Methods section.*
☒	☐	A description of all covariates tested
☐	☒	A description of any assumptions or corrections, such as tests of normality and adjustment for multiple comparisons
☐	☒	A full description of the statistical parameters including central tendency (e.g. means) or other basic estimates (e.g. regression coefficient) AND variation (e.g. standard deviation) or associated estimates of uncertainty (e.g. confidence intervals)
☐	☒	For null hypothesis testing, the test statistic (e.g. *F*, *t*, *r*) with confidence intervals, effect sizes, degrees of freedom and *P* value noted<br>*Give P values as exact values whenever suitable.*
☒	☐	For Bayesian analysis, information on the choice of priors and Markov chain Monte Carlo settings
☒	☐	For hierarchical and complex designs, identification of the appropriate level for tests and full reporting of outcomes
☐	☒	Estimates of effect sizes (e.g. Cohen's *d*, Pearson's *r*), indicating how they were calculated

Our web collection on statistics for biologists contains articles on many of the points above.

Software and code

Policy information about availability of computer code

Data collection	Hystar v6.0
Data analysis	DIA NN v.1.7.1, MSfragger 3.7, FragPipe 20, MSconvert v3.0.21072-998eff1c0 (developer build), XiSearch v1.7.6.7, Python 3.7, Tensorflow 2.12.0, biopython 1.8.0, EMBOSS v6.6.0.0, bedtools, PPIprophet (https://github.com/anfoss/PPIprophet)

For manuscripts utilizing custom algorithms or software that are central to the research but not yet described in published literature, software must be made available to editors and reviewers. We strongly encourage code deposition in a community repository (e.g. GitHub). See the Nature Portfolio guidelines for submitting code & software for further information.

Data

Policy information about availability of data

All manuscripts must include a data availability statement. This statement should provide the following information, where applicable:

- Accession codes, unique identifiers, or web links for publicly available datasets
- A description of any restrictions on data availability
- For clinical datasets or third party data, please ensure that the statement adheres to our policy

The supporting MS data is available via Massive with the identifier MSV000091715.
The PhageMap database is freely accessible at https://phagemap.ucsf.edu/
The Alphafold2 predicted structures are available on GitHub at https://github.com/anfoss/Phage_data and Zenodo at DOI: 10.5281/zenodo.8161692

Research involving human participants, their data, or biological material

Policy information about studies with human participants or human data. See also policy information about sex, gender (identity/presentation), and sexual orientation and race, ethnicity and racism.

Reporting on sex and gender	NA
Reporting on race, ethnicity, or other socially relevant groupings	NA
Population characteristics	NA
Recruitment	NA
Ethics oversight	NA

Note that full information on the approval of the study protocol must also be provided in the manuscript.

Field-specific reporting

Please select the one below that is the best fit for your research. If you are not sure, read the appropriate sections before making your selection.

☒ Life sciences ☐ Behavioural & social sciences ☐ Ecological, evolutionary & environmental sciences

For a reference copy of the document with all sections, see nature.com/documents/nr-reporting-summary-flat.pdf

Life sciences study design

All studies must disclose on these points even when the disclosure is negative.

Sample size	We employed duplicate samples as previously shown for SEC-MS datasets
Data exclusions	No data was excluded
Replication	Biological duplicates were performed and the data was successfully replicated
Randomization	No randomization was performed to maintain quantitative accuracy in MS measurements of successive fractions
Blinding	Blinding was not applicable due to the nature of SEC-MS data which requires to be run in the order determined by the fractionation scheme employed

Reporting for specific materials, systems and methods

We require information from authors about some types of materials, experimental systems and methods used in many studies. Here, indicate whether each material, system or method listed is relevant to your study. If you are not sure if a list item applies to your research, read the appropriate section before selecting a response.

Materials & experimental systems

n/a	Involved in the study	
☐	☒	Antibodies
☒	☐	Eukaryotic cell lines
☒	☐	Palaeontology and archaeology
☒	☐	Animals and other organisms
☒	☐	Clinical data
☒	☐	Dual use research of concern
☒	☐	Plants

Methods

n/a	Involved in the study	
☒	☐	ChIP-seq
☒	☐	Flow cytometry
☒	☐	MRI-based neuroimaging

Antibodies

Antibodies used	anti-FLAG M2 (Sigma F1804-50UG), Goat anti-Mouse igG (H+L) Secondary Antibody HRP (Ref #: 62-6520; Lot #: XD347166 Invitrogen)
Validation	We included positive and negative controls in the western blot. No independent Ab validation was performed
