# Supplementary figures and images for: Next-generation proteomics for quantitative Jumbophage-bacteria interaction mapping
Source: Nat Commun. 2023 Aug 24;14:5156. doi: 10.1038/s41467-023-40724-w (PMC10449902; doi:10.1038/s41467-023-40724-w)

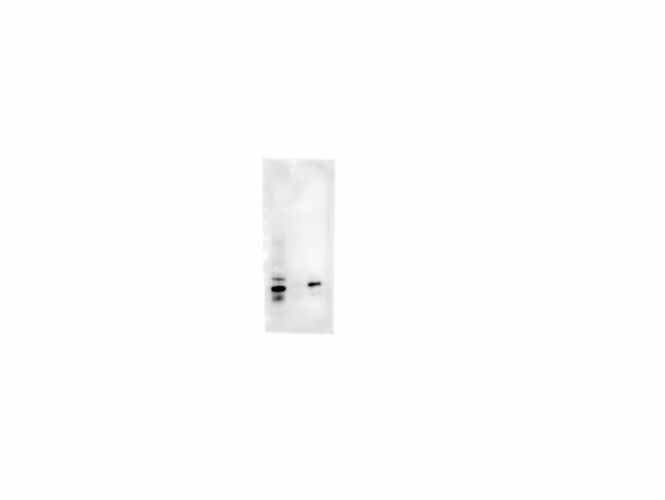

Supplement: Supplementary file 7 — Source Data [file 41467_2023_40724_MOESM7_ESM.zip › blots_cropped/PHIKZ184.tif]

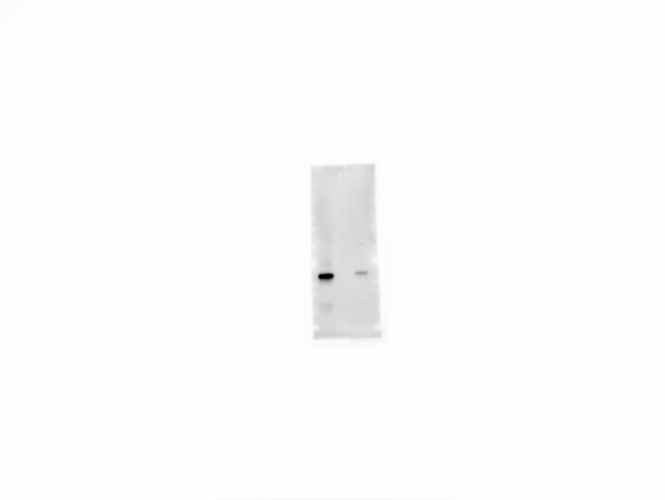

Supplement: Supplementary file 7 — Source Data [file 41467_2023_40724_MOESM7_ESM.zip › blots_cropped/PHIKZ153.tif]

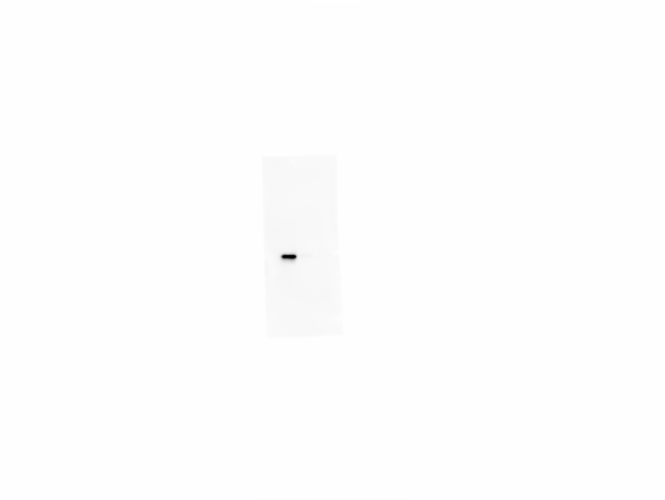

Supplement: Supplementary file 7 — Source Data [file 41467_2023_40724_MOESM7_ESM.zip › blots_cropped/PHIKZ030.tif]

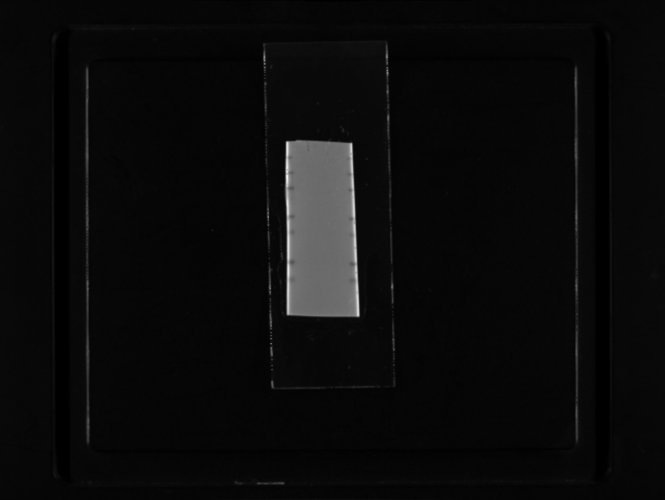

Supplement: Supplementary file 7 — Source Data [file 41467_2023_40724_MOESM7_ESM.zip › blots_cropped/PHIKZ177_Ladder.tif]

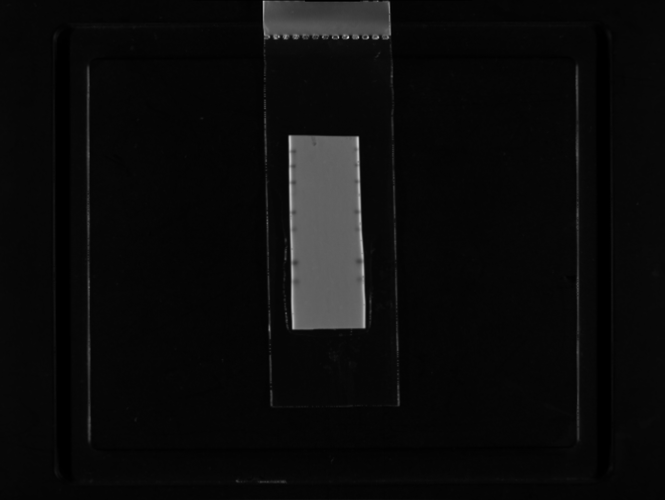

Supplement: Supplementary file 7 — Source Data [file 41467_2023_40724_MOESM7_ESM.zip › blots_cropped/PHIKZ163_Ladder.tif]

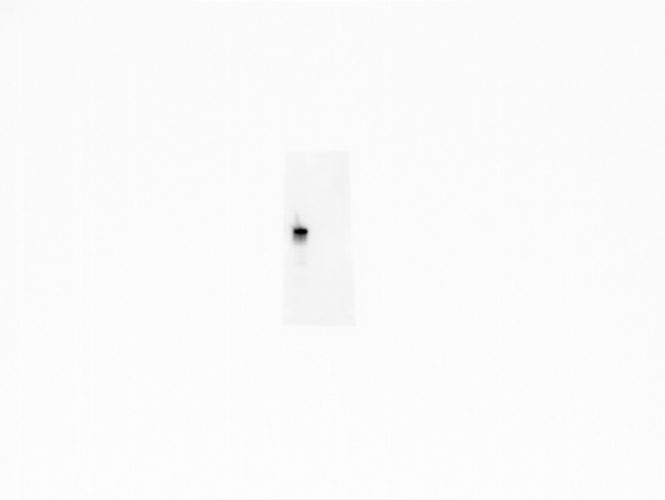

Supplement: Supplementary file 7 — Source Data [file 41467_2023_40724_MOESM7_ESM.zip › blots_cropped/PHIKZ157.tif]

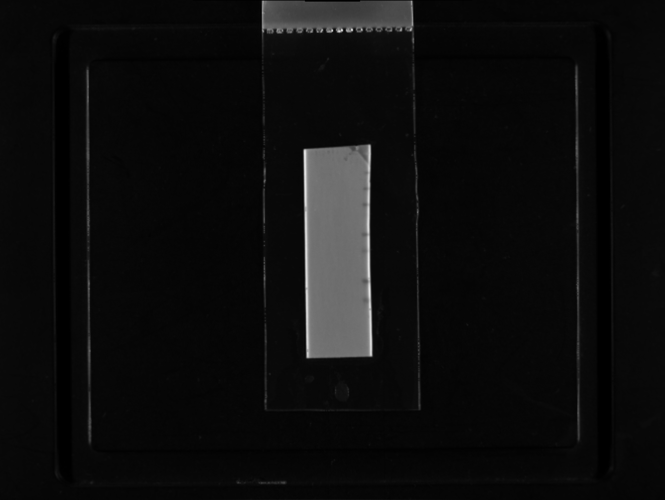

Supplement: Supplementary file 7 — Source Data [file 41467_2023_40724_MOESM7_ESM.zip › blots_cropped/PHIKZ094_Ladder.tif]

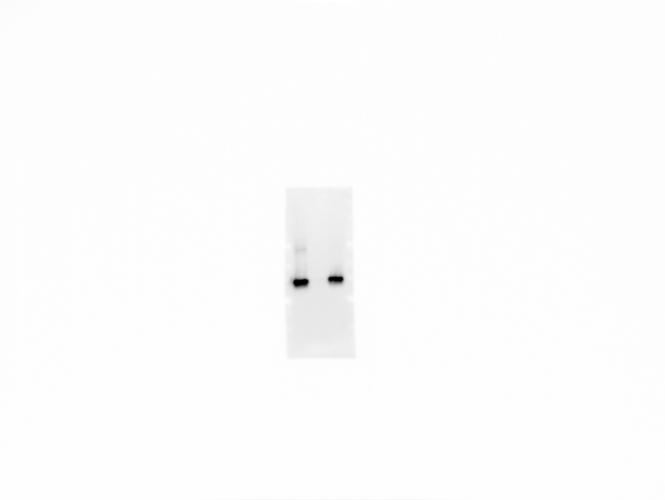

Supplement: Supplementary file 7 — Source Data [file 41467_2023_40724_MOESM7_ESM.zip › blots_cropped/PHIKZ093.tif]

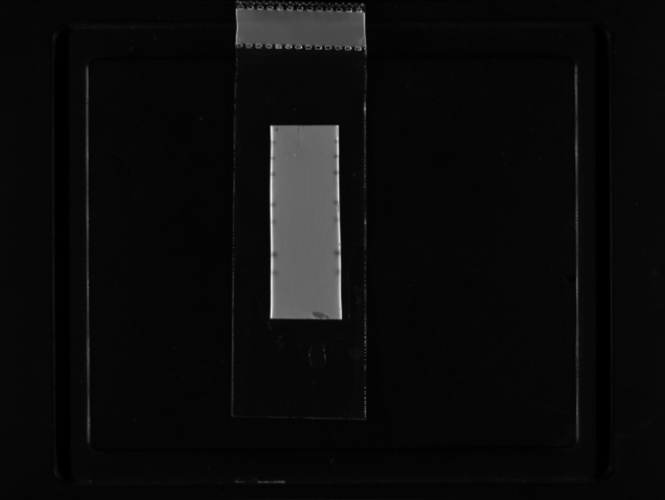

Supplement: Supplementary file 7 — Source Data [file 41467_2023_40724_MOESM7_ESM.zip › blots_cropped/PHIKZ244_Ladder.tif]

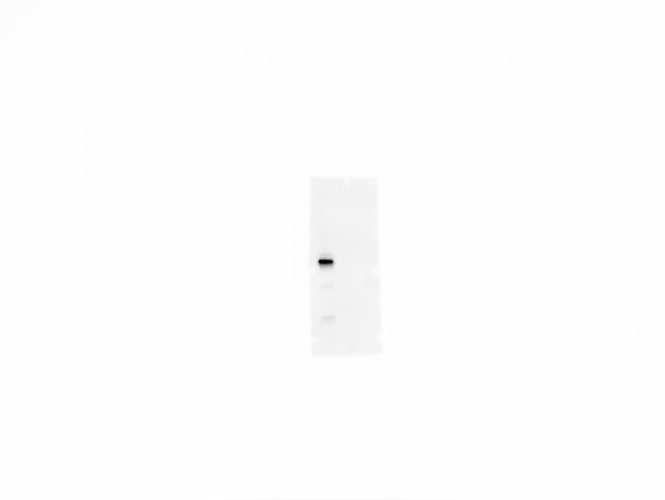

Supplement: Supplementary file 7 — Source Data [file 41467_2023_40724_MOESM7_ESM.zip › blots_cropped/PHIKZ092.tif]

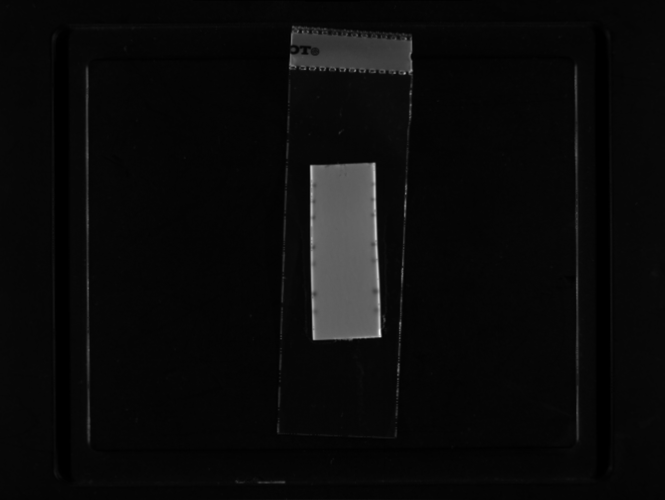

Supplement: Supplementary file 7 — Source Data [file 41467_2023_40724_MOESM7_ESM.zip › blots_cropped/PHIKZ153_Ladder.tif]

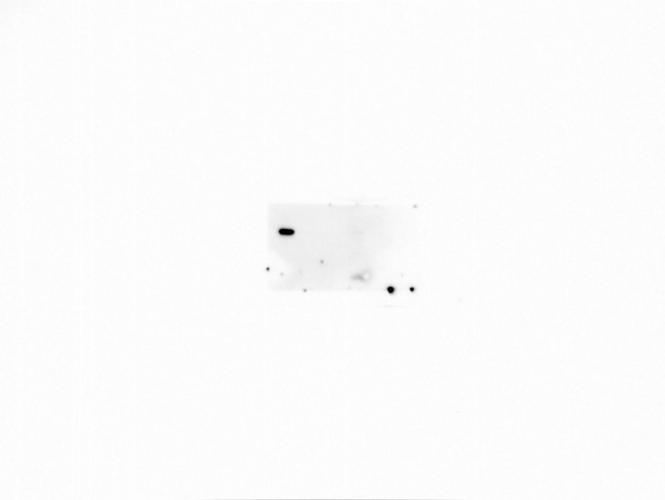

Supplement: Supplementary file 7 — Source Data [file 41467_2023_40724_MOESM7_ESM.zip › blots_cropped/PHIKZ090.tif]

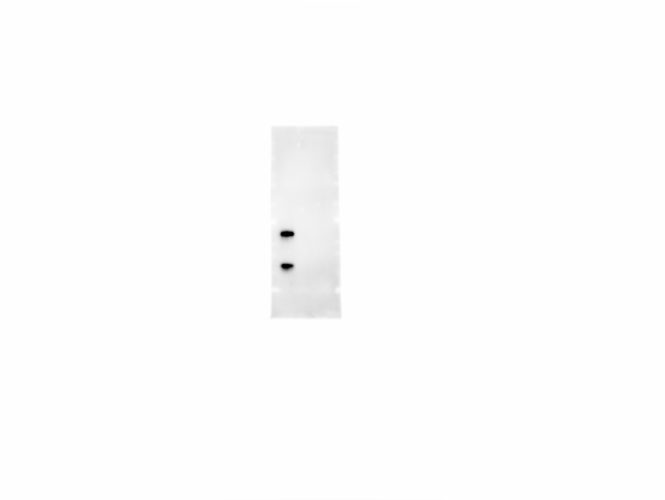

Supplement: Supplementary file 7 — Source Data [file 41467_2023_40724_MOESM7_ESM.zip › blots_cropped/PHIKZ244.tif]

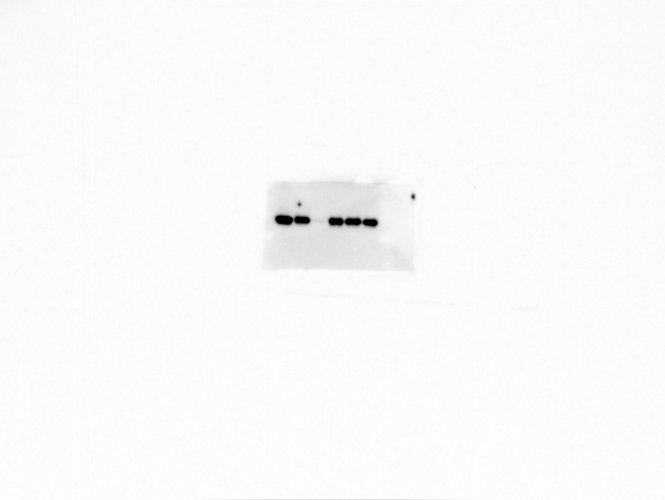

Supplement: Supplementary file 7 — Source Data [file 41467_2023_40724_MOESM7_ESM.zip › blots_cropped/PHIKZ095.tif]

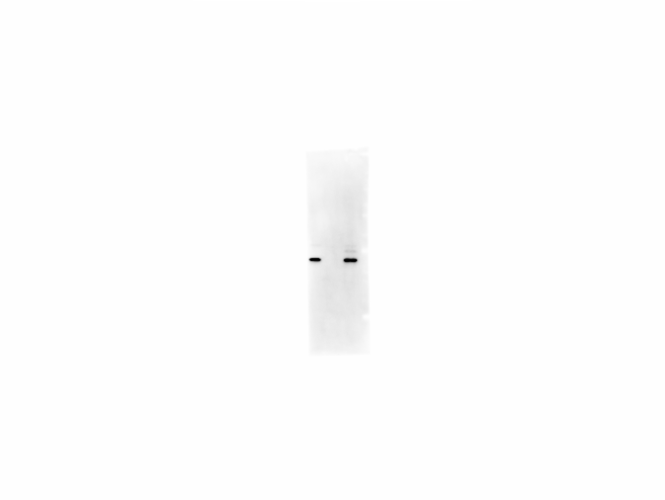

Supplement: Supplementary file 7 — Source Data [file 41467_2023_40724_MOESM7_ESM.zip › blots_cropped/PHIKZ094.tif]

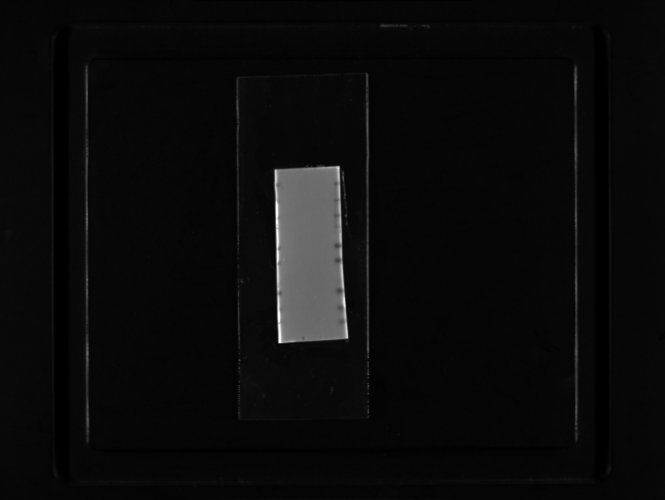

Supplement: Supplementary file 7 — Source Data [file 41467_2023_40724_MOESM7_ESM.zip › blots_cropped/PHIKZ089_Ladder.tif]

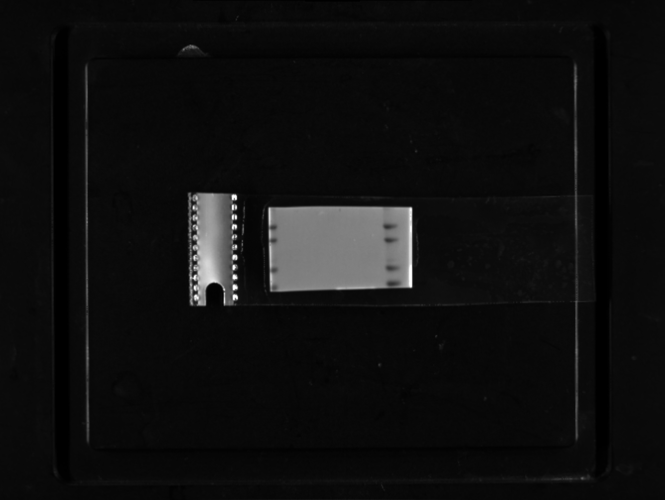

Supplement: Supplementary file 7 — Source Data [file 41467_2023_40724_MOESM7_ESM.zip › blots_cropped/PHIKZ162_Ladder.tif]

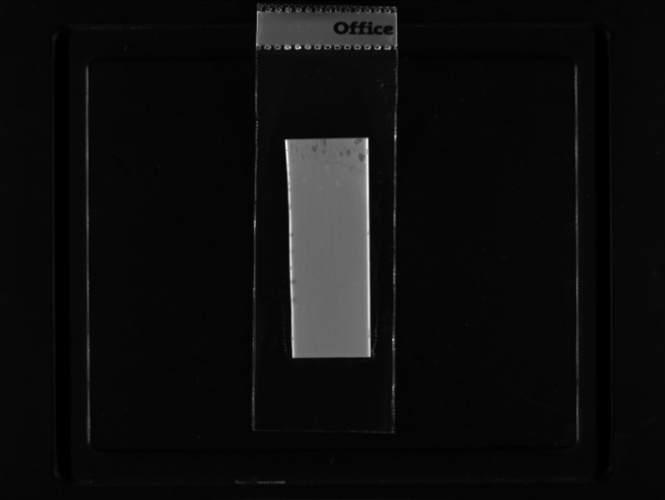

Supplement: Supplementary file 7 — Source Data [file 41467_2023_40724_MOESM7_ESM.zip › blots_cropped/PHIKZ303_Ladder.tif]

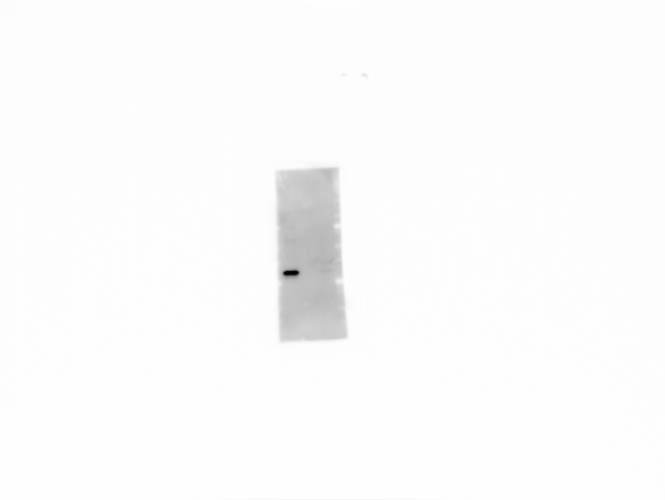

Supplement: Supplementary file 7 — Source Data [file 41467_2023_40724_MOESM7_ESM.zip › blots_cropped/PHIKZ089.tif]

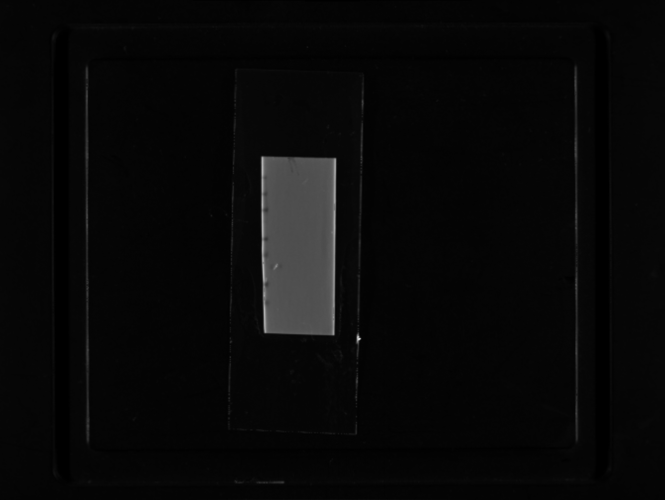

Supplement: Supplementary file 7 — Source Data [file 41467_2023_40724_MOESM7_ESM.zip › blots_cropped/PHIKZ184_Ladder.tif]

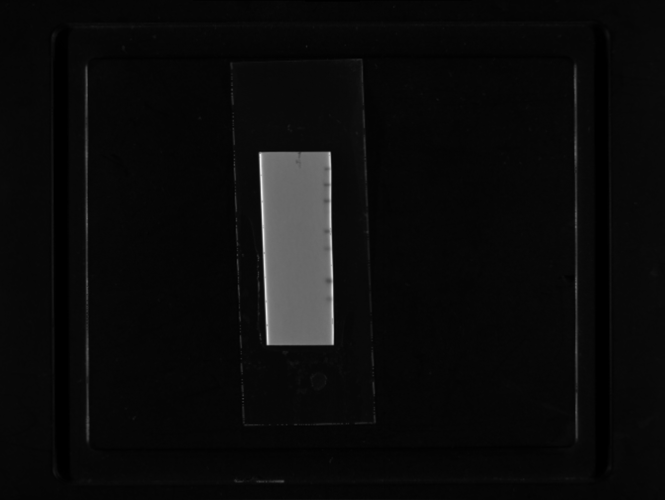

Supplement: Supplementary file 7 — Source Data [file 41467_2023_40724_MOESM7_ESM.zip › blots_cropped/PHIKZ203_Ladder.tif]

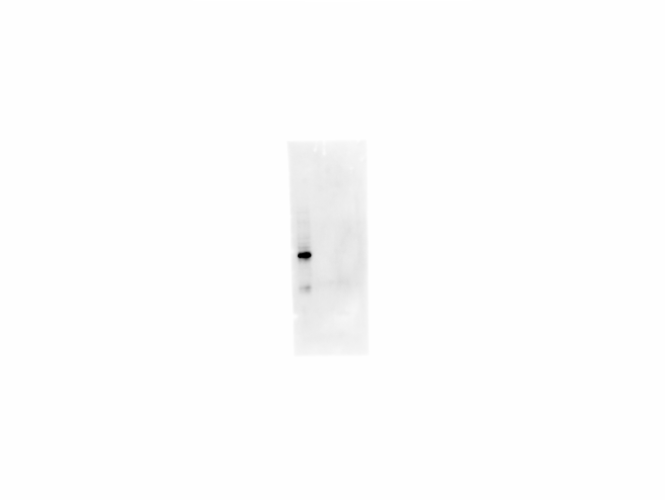

Supplement: Supplementary file 7 — Source Data [file 41467_2023_40724_MOESM7_ESM.zip › blots_cropped/PHIKZ303.tif]

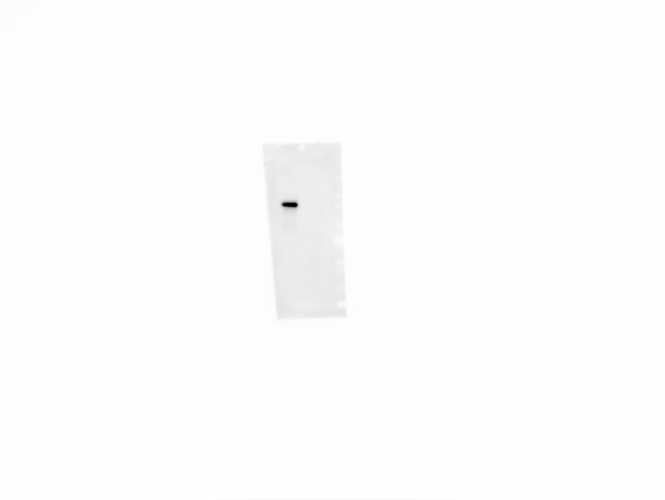

Supplement: Supplementary file 7 — Source Data [file 41467_2023_40724_MOESM7_ESM.zip › blots_cropped/PHIKZ129.tif]

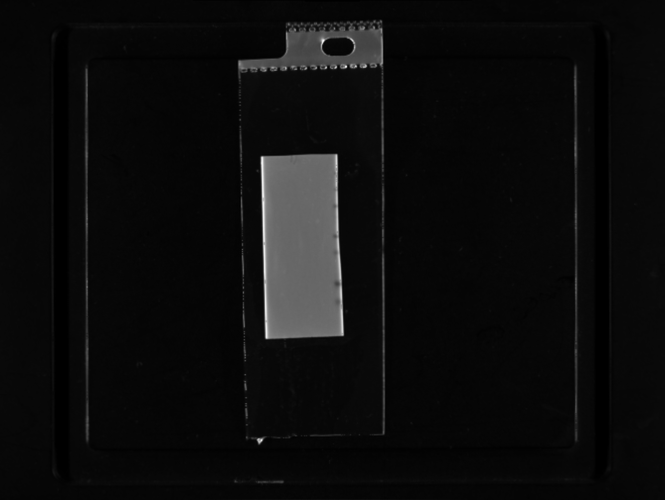

Supplement: Supplementary file 7 — Source Data [file 41467_2023_40724_MOESM7_ESM.zip › blots_cropped/PHIKZ030_Ladder.tif]

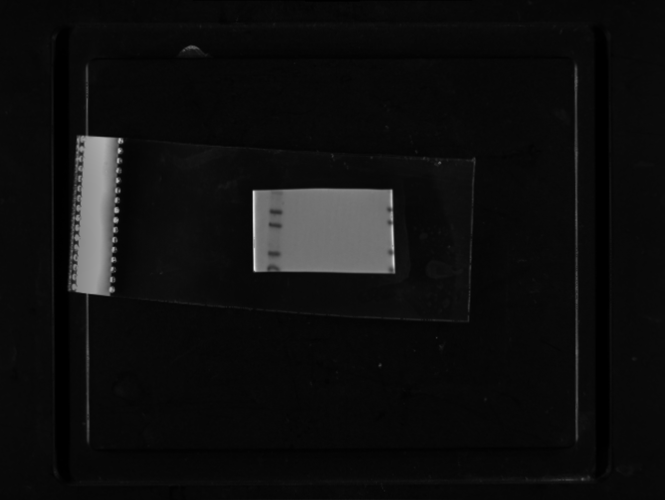

Supplement: Supplementary file 7 — Source Data [file 41467_2023_40724_MOESM7_ESM.zip › blots_cropped/PHIKZ097_Ladder.tif]

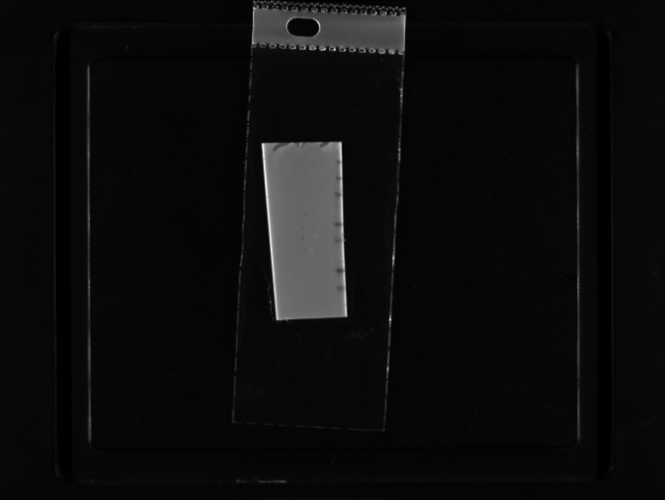

Supplement: Supplementary file 7 — Source Data [file 41467_2023_40724_MOESM7_ESM.zip › blots_cropped/PHIKZ129_Ladder.tif]

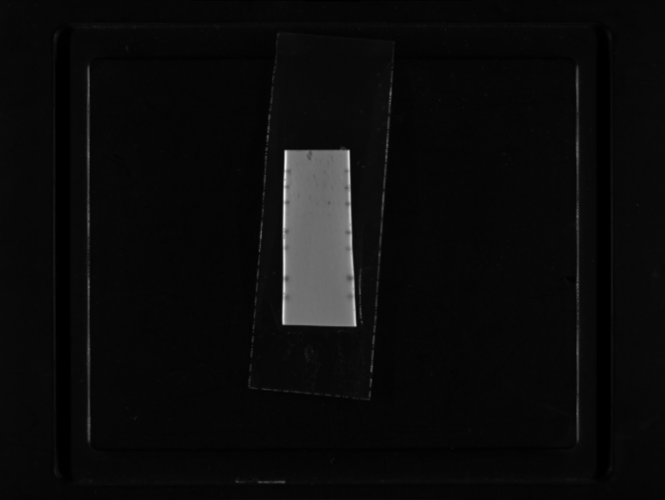

Supplement: Supplementary file 7 — Source Data [file 41467_2023_40724_MOESM7_ESM.zip › blots_cropped/PHIKZ157_Ladder.tif]

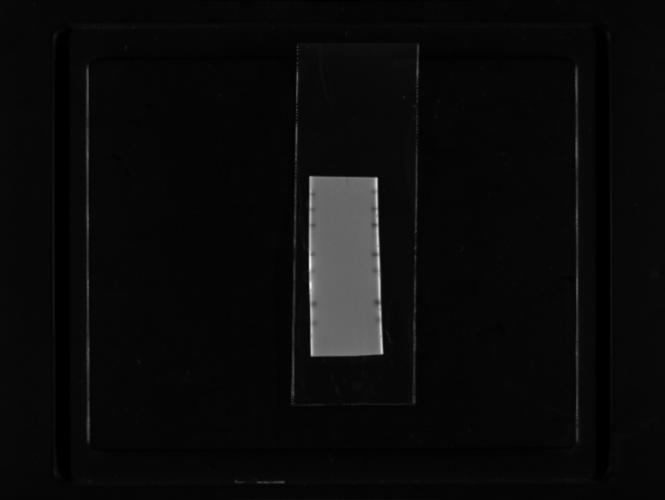

Supplement: Supplementary file 7 — Source Data [file 41467_2023_40724_MOESM7_ESM.zip › blots_cropped/PHIKZ092_Ladder.tif]

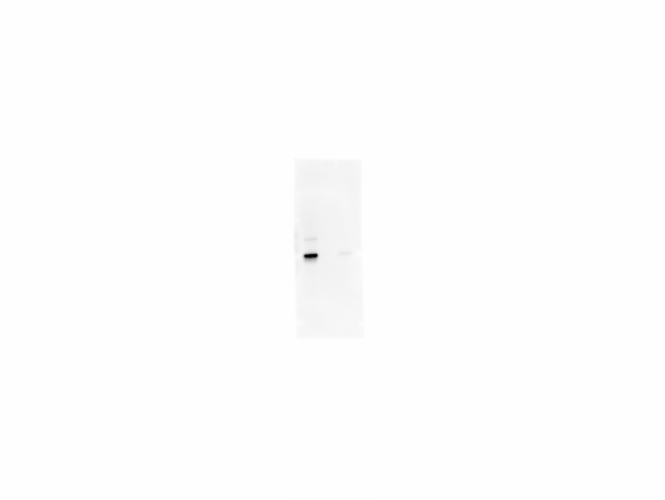

Supplement: Supplementary file 7 — Source Data [file 41467_2023_40724_MOESM7_ESM.zip › blots_cropped/PHIKZ086 (p29).tif]

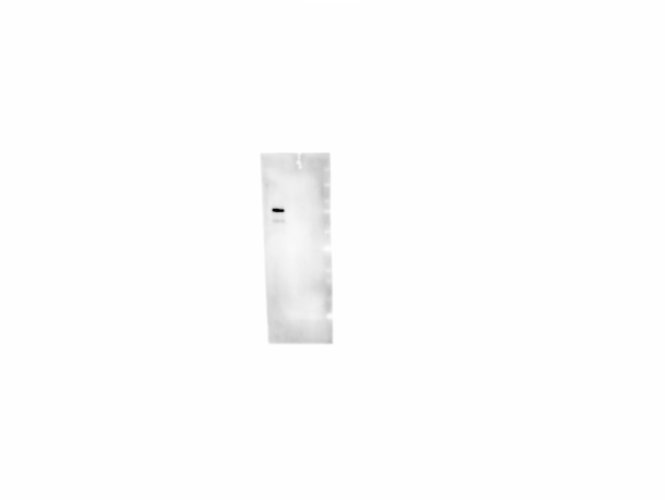

Supplement: Supplementary file 7 — Source Data [file 41467_2023_40724_MOESM7_ESM.zip › blots_cropped/PHIKZ203.tif]

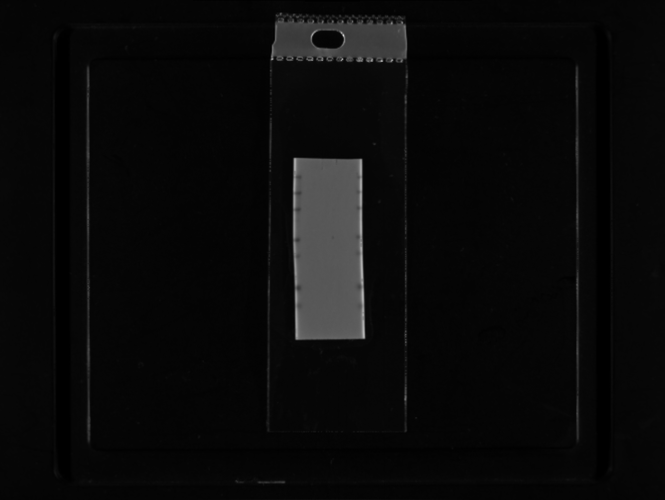

Supplement: Supplementary file 7 — Source Data [file 41467_2023_40724_MOESM7_ESM.zip › blots_cropped/PHIKZ086 (p29)_Ladder.tif]

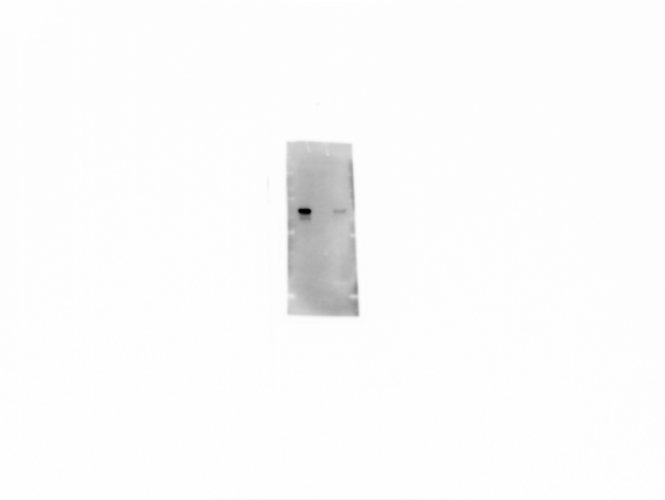

Supplement: Supplementary file 7 — Source Data [file 41467_2023_40724_MOESM7_ESM.zip › blots_cropped/PHIKZ177.tif]

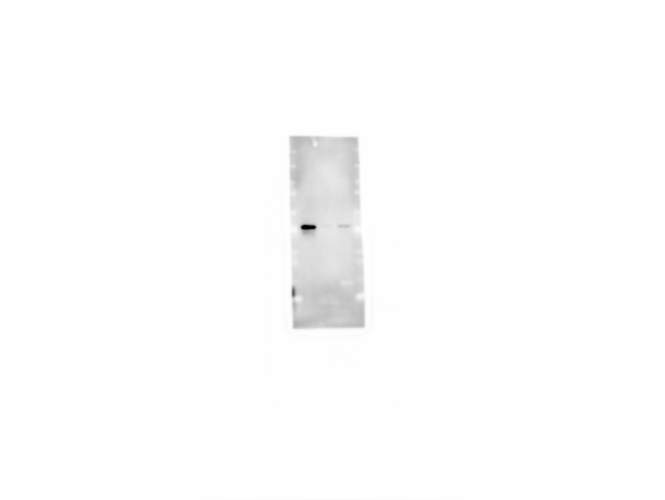

Supplement: Supplementary file 7 — Source Data [file 41467_2023_40724_MOESM7_ESM.zip › blots_cropped/PHIKZ163.tif]

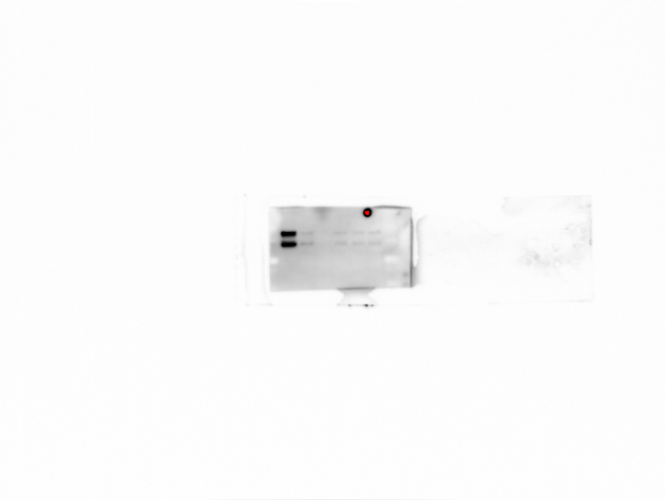

Supplement: Supplementary file 7 — Source Data [file 41467_2023_40724_MOESM7_ESM.zip › blots_cropped/PHIKZ162.tif]
